# Vivisection

**Published:** 1874-12

**Authors:** 


### Vivisection.

At a meeting of the New York Pathological Society, held Nov., 12th, 1874, the following preamble and resolutions were unanimously adopted:

*Whereas*, Certain publications have recently been brought to the notice of this Society, having for their object to excite a prejudice against the propriety and utility of experiments upon living animals, as performed for the purpose of medical investigation, and to control or prohibit such experiments by legal enactment; and

*Whereas*, The members of this Society have abundant reason to know that the method of experimentation upon animals has been productive of many useful discoveries in pathology, as well as in physiology and practical medicine; therefore,

RESOLVED, I. That experimentation upon animals is of the same value for investigation and discovery in medicine as experiments in physics and chemistry for the corresponding branches of Natural Science;

II. That this method of investigation, by increasing our knowledge of the causes and consequences of disease, leads to enlarged facilities for its prevention and cure, and has thus been the means, in many instances of preserving human life, and relieving human suffering:

III. That, in the opinion of this Society, it would be in the highest degree impolitic and unjust to prohibit experimental investigations of this nature, or to withdraw from them the protection and recognition which is wisely afforded by the law.

Unanimously adopted.

H. Knapp, M. D. President.

Geo. F. Shrady, M. D. Secretary.

*Medical Record.*

